# Fluorescent membrane potential assay for drug screening on Kv10.1 channel: identification of BL-1249 as a channel activator

**DOI:** 10.3389/fphar.2023.1238503

**Published:** 2023-07-24

**Authors:** Mirsha Aseret Gómez-Herrera, Enikar Patlán, Armando Estrada-Garrido, Arturo Hernández-Cruz, Enoch Luis

**Affiliations:** ^1^ Laboratorio Nacional de Canalopatías, Instituto de Fisiología Celular, Universidad Nacional Autónoma de México, Mexico City, Mexico; ^2^ Universidad Tecnológica de México (UNITEC)—Campus Ecatepec, Estado de México, Mexico; ^3^ Cátedras CONAHCYT—Instituto de Fisiología Celular, Universidad Nacional Autónoma de México, Mexico City, Mexico

**Keywords:** membrane potential, Kv10.1, oncochannel, Kv opener, cell-based fluorescent assay

## Abstract

Resting membrane potential is a bioelectric property of all cells. Multiple players govern this property, the ion channels being the most important. Ion channel dysfunction can affect cells’ resting membrane potential and could be associated with numerous diseases. Therefore, the drug discovery focus on ion channels has increased yearly. In addition to patch-clamp, cell-based fluorescent assays have shown a rapid and reliable method for searching new ion channel modulators. Here, we used a cell-based membrane potential assay to search for new blockers of the Kv10.1, a potassium channel strongly associated with cancer progression and a promising target in anticancer therapy. We found that fluoxetine and miconazole can inhibit the Kv10.1 channel in the micromolar range. In contrast, BL-1249 potentiates Kv10.1 currents in a dose-dependent manner, becoming the first molecule described as an activator of the channel. These results demonstrate that cell-based membrane potential assay can accelerate the discovery of new Kv10.1 modulators.

## 1 Introduction

The resting membrane potential (RMP) is a bioelectric property that governs many cellular processes in physiological and pathological conditions (Abdul Kadir et al., 2018). This cell property is mainly governed by the activity of diverse voltage-gated ion channels, which can rapidly rearrange charges (ions) close to the plasma membrane (Curtis and Cole, 1942). The opening and closing of these ion channels can influence the resting membrane potential, making it more hyperpolarized or depolarized. For decades, patch-clamp electrophysiology has been the most accurate method for characterizing the function of ion channels and correlating their activity with bioelectric phenomena (Neher and Sakmann, 1976; Verkhratsky and Parpura, 2014). Nevertheless, the development of high-throughput fluorescent systems and improvements in fluorescent dyes have made it possible to study cellular phenomena, such as the RMP, quickly and correctly (Vetter et al., 2020; Loza-Huerta et al., 2021). For fluorescence membrane potential assays (e.g., FLIPR^®^ membrane potential), some negatively charged dyes incorporate into the membrane lipid of the cells and increase their fluorescence intensity upon depolarization (Vetter et al., 2020).

Since 1999, the voltage-gated potassium channel Kv10.1, also named Eag1, has emerged as an exciting target in cancer therapy (Pardo et al., 1999). Kv10.1 belong to the superfamily of voltage-gated potassium channels and is assembled as homotetramers (Whicher and MacKinnon, 2016). Diverse authors have shown an overexpression of Kv10.1 in cancer samples, contrasting with a low expression in healthy tissues (Farias et al., 2004; Hemmerlein et al., 2006; Ortiz et al., 2011; Martínez et al., 2015; Luis et al., 2022a). Also, it has been described that Kv10.1 overexpression is associated with increased cell proliferation, angiogenesis, and chemo- and radio-resistance phenotype (Pardo et al., 1999; Downie et al., 2008; Bai et al., 2013; Luis et al., 2022b). Notably, Kv10.1 knock-out mice show no behavioral abnormalities, suggesting that side effects due to the Kv10.1-blocking can be insignificant. All this evidence and their transmembrane nature make Kv10.1 a promising target for drug development and screening in the cancer biology context.

Here, we validate a primary screening using the cell-based FLIPR^®^ membrane potential (FMP) for searching new Kv10.1 modulators. Most of the molecules tested here were supplied from Alomone labs’ ion channel modulator explorer kits, which were selected based on their know effect on other potassium channels, e.g., the Kv11.1. Thus, we found that fluoxetine, miconazole nitrate (MN), and BL-1249 (BL) modulate the fluorescents signals associated with Kv10.1 activity. Patch-clamp recordings corroborate FMP results and showed that the three molecules had dose-dependent effects on Kv10.1 channels, with fluoxetine and MN having inhibitory and BL opener activity. These results demonstrate that FMP can produce reliable results and accelerate the discovery of new Kv10.1 modulators.

## 2 Methods

### 2.1 Cell cultures

HEK293 wild-type cells (HEK-WT; CRL-1573, ATCC) and HEK293 cells stably expressing the human Kv10.1 potassium channel (HEK-Kv10.1) (generously provided by Dr. Walter Stühmer from the Max Plack Institute) were cultured in Dulbecco’s Modified Eagle Medium (DMEM) (12800-017, Gibco) containing 10% fetal bovine serum (26140087, Gibco) and 1% Penicillin/Streptomycin (15140122, Gibco). HEK-Kv10.1 were supplemented with Zeocin (30 μg/mL) (R25001, Invitrogen) as a selection antibiotic. All cells were cultured at 37°C in a 5% CO_2_ incubator.

### 2.2 Fluorescence membrane potential assay

FLIPR^®^ membrane potential assay BLUE (R8034, Molecular Devices) was performed following the manufacturer’s protocols. Flat-bottom 96-well plates (3,599, Costar) were plated with HEK-WT or HEK293-Kv10.1 at a density of 20,000 cells/well in 100 µL of supplemented DMEM and incubated for 24 h at 37°C in a 5% CO_2_ incubator. After 24 h, 100 µL of the FMP buffer was added for 30 min at 37°C in a 5% CO_2_ incubator; at this point, the molecules of interest were mixed in the FMP buffer and pre-incubated in the selected wells. All the molecules were tested at 100 μM; except astemizole, which was tested at 50 µM and DMSO (0.1%). After pre-incubation, 96-well plates were transferred to a FlexStation3 microplate reader (Molecular Devices) controlled by the SoftMax Pro 7 software. FLIPR dye was excited at 530 nm, and the emitted fluorescence was recorded at 565 nm. Data were acquired at 0.5 Hz for 120 s: the first 20 s represent the basal fluorescence, and then, 50 µL (5x) of the high potassium solution was added (that resulted in a final K^+^ concentration of 60 mM), and the fluorescence signal was recorded for another 100 s. The fluorescence responses were normalized by F=F/F_0_, where F represents the fluorescence at any given time, and F_0_ is the mean basal fluorescence obtained in the first 20 s of recording. Once normalized, the amplitude was calculated as the difference between the basal fluorescence and the maximal amplitude at 120 s. The maximal rate of fluorescence increase is obtained on the first derivative of the fluorescence signal.

### 2.3 Electrophysiology

Cells were plated on 18 mm circular glass coverslips previously treated with poly-l-lysine for patch-clamp recordings. Coverslips were transferred to a recording chamber (RC-26G, Warner Instruments, Hamden, CT, United States), and whole-cell voltage- and current-clamp recordings were performed at room temperature and under continuous perfusion (at a flow rate of 2 mL/min) with a standard bath solution. The standard bath solution contained (in mM): 137 NaCl, 5.4 KCl, 2 CaCl_2_, 1.3 MgCl_2_, 10 HEPES, and 10 D-glucose (300 mOsm/kg, pH 7.4 adjusted with NaOH). Intracellular patch-pipettes had resistances of 2–3 MΩ and were filled with an internal solution containing (in mM): 140 KCl, 1 MgCl_2_, 10 EGTA, 10 HEPES (300 mOsm/kg, pH 7.2 adjusted with KOH). Ionic currents were recorded with a setup composed of Multiclamp 700B/Digidata 1,550/pCamp10 (amplifier/analog-digital converter/software, all from Molecular Devices), filtered at 5 kHz, and digitally sampled at 10 kHz. 40%–60% of the series resistance was electronically compensated. Drugs tested on Kv10.1 currents were evaluated from a holding voltage (Vh) of −70 mV, and currents were evoked with 250 ms duration voltage steps from −70 to +30 mV (for BL-1249) or +50 mV (for fluoxetine and miconazole) applied every 5 s. I-V curves were constructed using a protocol of 250 ms voltage steps from −100 mV to +50 mV in 10 mV increments and a Vh of −70 mV; the amplitude of Kv10.1 currents was measured at the end of voltage steps (average of the last 3 ms) and plotted *versus* voltage. Conductance was assessed as G = I_K_/(V-V_K_), where V_K_ is the reversal potential for K^+^ flux, I_K_ is the Kv10.1 current, and V is the membrane potential. The corresponding normalized G (G/Gmax) is plotted *versus* voltage; data were fit in Origin 2019 with a single Boltzmann distribution: G/Gmax = 1/(1+e^−(V−V^1/2^)/s^), where V1/2 and s are the arithmetic means of half-maximal activation potentials and slope factors, respectively.

We performed whole-cell current clamp recording in the gap-free mode to test the effect of BL-1249 on the resting membrane potential of HEK-WT and HEK-Kv10.1 cells. Cells were held without current injection (0 pA), and the protocol consisted of 60 s in the standard bath solution, followed by 60 s in BL-1249 or DMSO, and 60 s for washout.

Concentration-inhibitory curves were fitted with the Hill equation using the Levenberg–Marquardt method implemented in Origin 2019 software: Inhibition = Bmax*c^n^/(IC50^n^ + c^n^), where Bmax is the maximum block, IC50 is the concentration of half-maximal inhibition, c is the concentration of the molecules, and n is the Hill coefficient.

For BL-1249, the concentration-effect curve was fitting using the method implemented in GraphPad Prism 8 software: Potentiation = 100/(1 + 10^(LogEC50−X)^), where EC50 is the concentration of half-maximal potentiation. This model assumes data is normalized, forcing the curve to run from 0% to 100%.

### 2.4 Molecules

Amitriptyline (A-155), bupivacaine hydrochloride (B-125), celecoxib (C-190), citalopram (C-195), flupirtine maleate (F-150), fluoxetine hydrochloride (F-155), ICA-11081 (I-160), L-ascorbic acid (L-140), linopiridine dihydrochloride (L-156), loperamide hydrochloride (L-100), miconazole nitrate (M-206), rosiglitazone (R-125) and 2,5-dimethylcelecoxib (D-150) were selected of a screening package for ion channel from Alomone Labs. BL-1249 (B2186), astemizole (A2861), and DMSO (D2650) were purchased from Sigma-Aldrich. Most stock solutions were prepared in 100% DMSO or water at a concentration of 100 mM. All molecules were diluted in a standard bath solution before starting every experiment. In patch-clamp experiments, drugs were delivered using a perfusion system VC-6 coupled to a fast-step solution charger (SF-77B, Warner Instruments, Hamden, CT, United States).

### 2.5 Statistical analysis

All data were analyzed using Origin 2019 (OriginLab, United States) and GraphPad Prism 8 (GraphPad Software, United States). Results are presented as mean ± SEM of at least three independent experiments. When two means were compared, statistical significance (*p* < 0.05) was assessed by Student’s t-test. For multiple comparisons, statistical significance (*p* < 0.05) was assessed by 1-way analysis of variance using the Dunnet *post hoc* test. Significant differences are expressed in figures: *p < 0.05, **p < 0.01, ***p < 0.001.

## 3 Results

### 3.1 FLIPR assay

**FIGURE 1:**
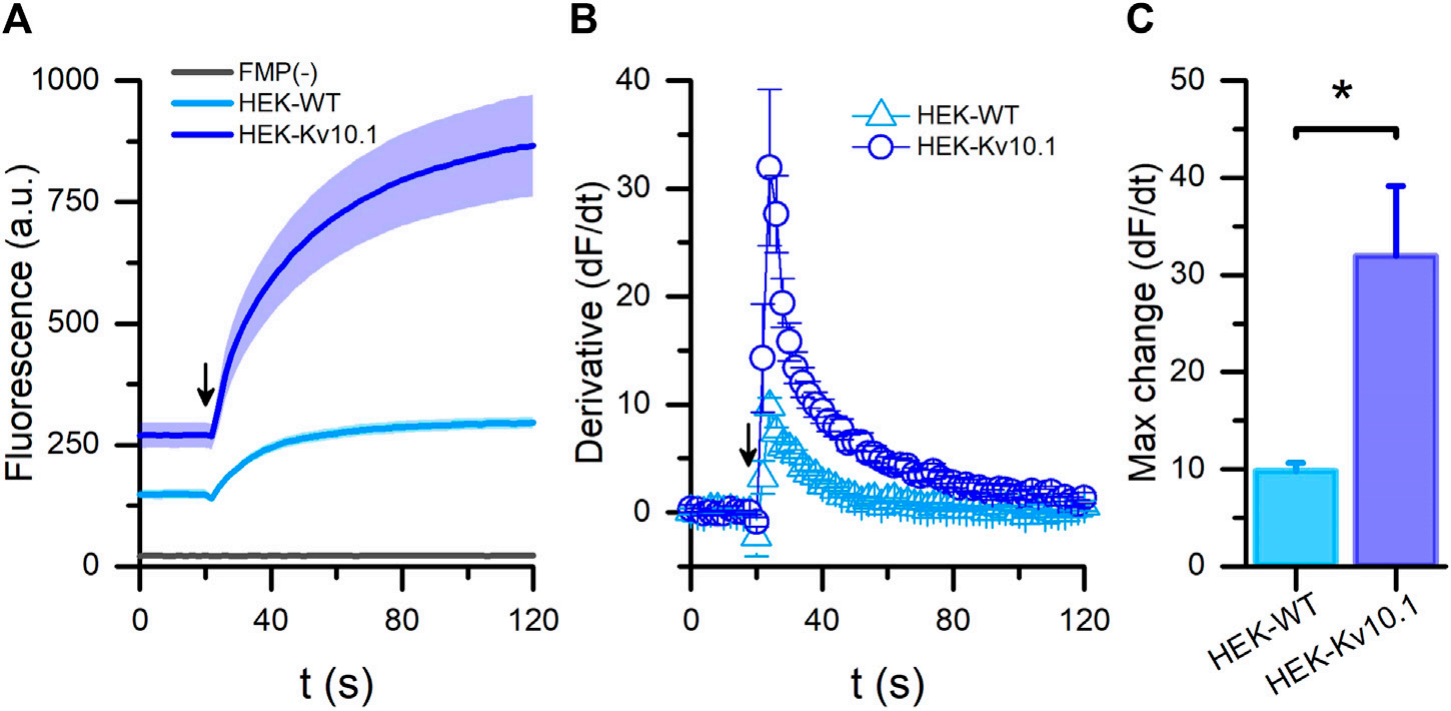
FLIPR^®^ membrane potential assay for Kv10.1 channel activity. **(A)** Membrane potential recordings in HEK-WT (cyan) and HEK-Kv10.1 (blue) cells using the FLIPR^®^-blue formulation. Arrow indicates when the high potassium (60 mM) stimulus was added to cells. HEK-Kv10.1 cells without any dye (FMP(-); black line) showed no fluorescent signal. The solid traces and faded colors show the mean ± SEM, respectively. **(B)** First derivative of the fluorescents signal showed that high potassium solutions induce faster signals in HEK-Kv10.1 than HEK-WT cells. **(C)** Comparison of the maximum fluorescence change in the different cells evaluated.

First, we tested the fluorescence responses to depolarization from HEK-WT and HEK-Kv10.1 cells using the FMP BLUE formulation. Adding a high K^+^ solution increased the fluorescence signals in both cell lines. Nonetheless, the fluorescence intensity recorded at 120 s in HEK-Kv10.1 cells was three times larger than in the HEK-WT, probably due to the expression of Kv10.1 channels (Figure 1A; *p* = 0.0006, unpaired *t*-test). Using the first derivative of the fluorescence responses, we observed that the maximum rate of change in HEK-Kv10.1 cells was around three times faster than in HEK-WT (Figures 1B, C; *p* = 0.0391, unpaired *t*-test). HEK-Kv10.1 cells that were not incubated with the FMP dye did not show a fluorescence signal either during the basal recording or the stimulation with a high potassium solution (Figure 1A). These results indicated that FMP assays are appropriate for studying Kv10.1 channel activity. Before starting the pharmacological screening, we evaluated the RMP on wild-type and Kv10.1-expressing cells. We measured the RMP in I = 0 mode within 30 s of achieving the whole cell configuration in patch-clamp, and recordings showed that HEK-WT cells have more depolarized values than HEK-Kv10.1, of −5.7 ± 1.0 mV (n = 31) and −44.4 ± 1.5 mV (n = 78), respectively (*p* < 0.001, unpaired *t*-test).

**FIGURE 2:**
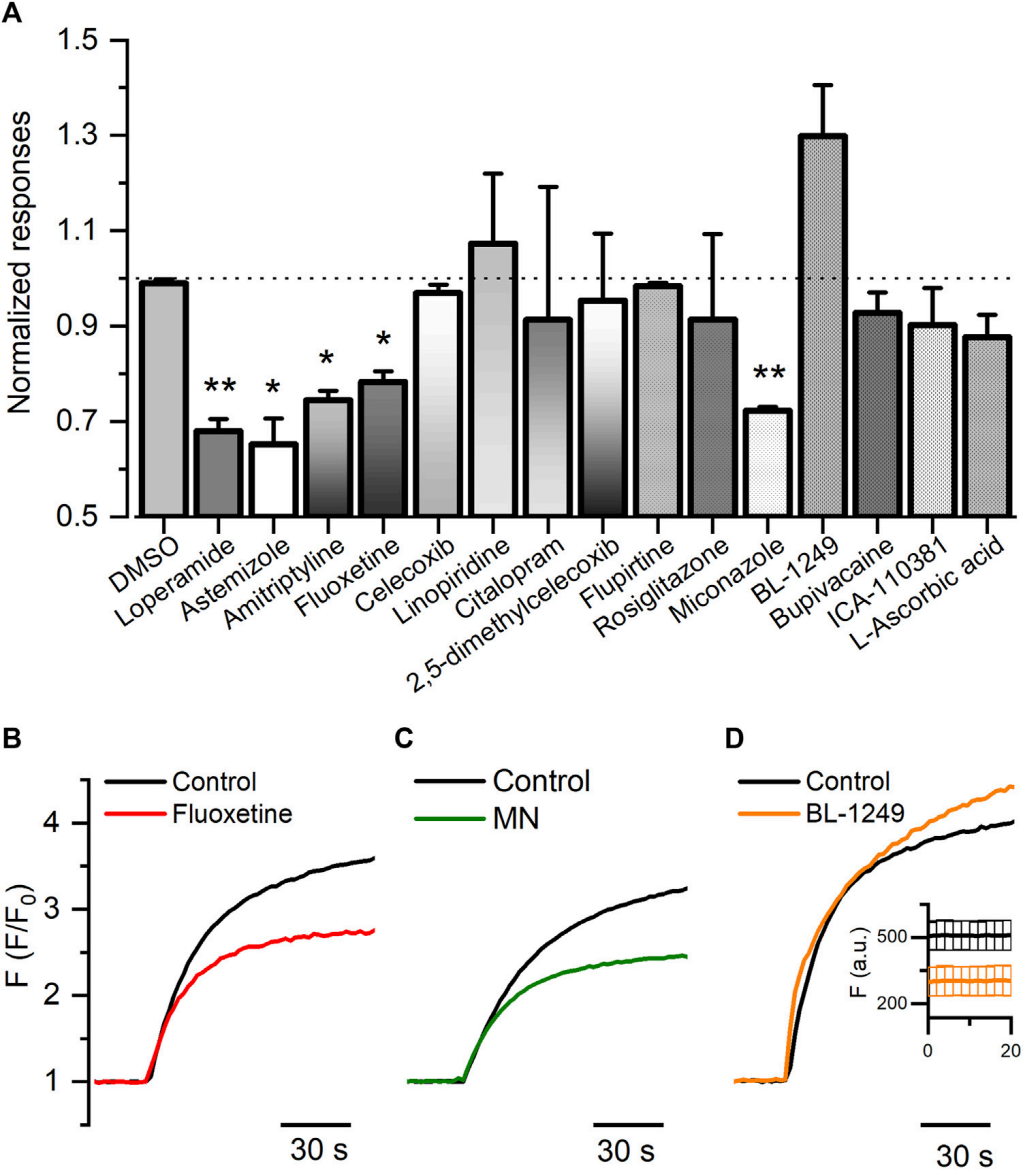
FLIPR^®^ membrane potential for HEK-Kv10.1 cells. **(A)** Summary bar graph of the fluorescence responses in HEK-Kv10.1 cell in the presence of different small molecules. Data were normalized to the control wells. Each bar represents the mean ± SEM of at least three independent experiments. **(B–D)** Representative recordings of the effect of fluoxetine, miconazole, and BL-1249 on the Kv10.1-FMP responses, respectively. In D, the insert represents the changes in the basal fluorescence in control (black) and the presence of BL-1249.

To validate the FMP assay, we pre-incubated HEK-Kv10.1 with previously described channel blockers, including astemizole, loperamide, and amitriptyline. As expected, astemizole, loperamide, and amitriptyline significantly decreased the fluorescence responses induced by the high potassium depolarization by 34.8%, 31.9%, and 25.5%, respectively (Figure 2A) (*p* = 0.0394, 0.0054, and 0.0192, respectively, 1-way ANOVA). DMSO (0.1%) did not produce any change in the responses (Figure 2A) (*p* = 0.8274, 1-way ANOVA). These results confirmed the method’s reliability. Next, we evaluated 12 small molecules and found that fluoxetine and miconazole nitrate significantly decreased the responses by 21.7% and 27.7%, respectively (Figures 2A–C) (*p* = 0.0361, and 0.0028, respectively, 1-way ANOVA). Interestingly, BL-1249 seems to increase the Kv10.1-mediated responses by 30% (Figure 2A); however, this effect was not statistically significant. Nevertheless, when we observed the basal responses (before normalization), we noticed that the basal fluorescence decreased statistically in the presence of BL (insert on Figure 2D) (*p* = 0.0189), suggesting BL could have activity on Kv10.1 channels.

### 3.2 Fluoxetine and miconazole inhibit the Kv10.1 channels

Next, we performed whole-cell voltage-clamp recordings in HEK-Kv10.1 cells to determine whether fluoxetine and miconazole act on Kv10.1 channels.

**FIGURE 3:**
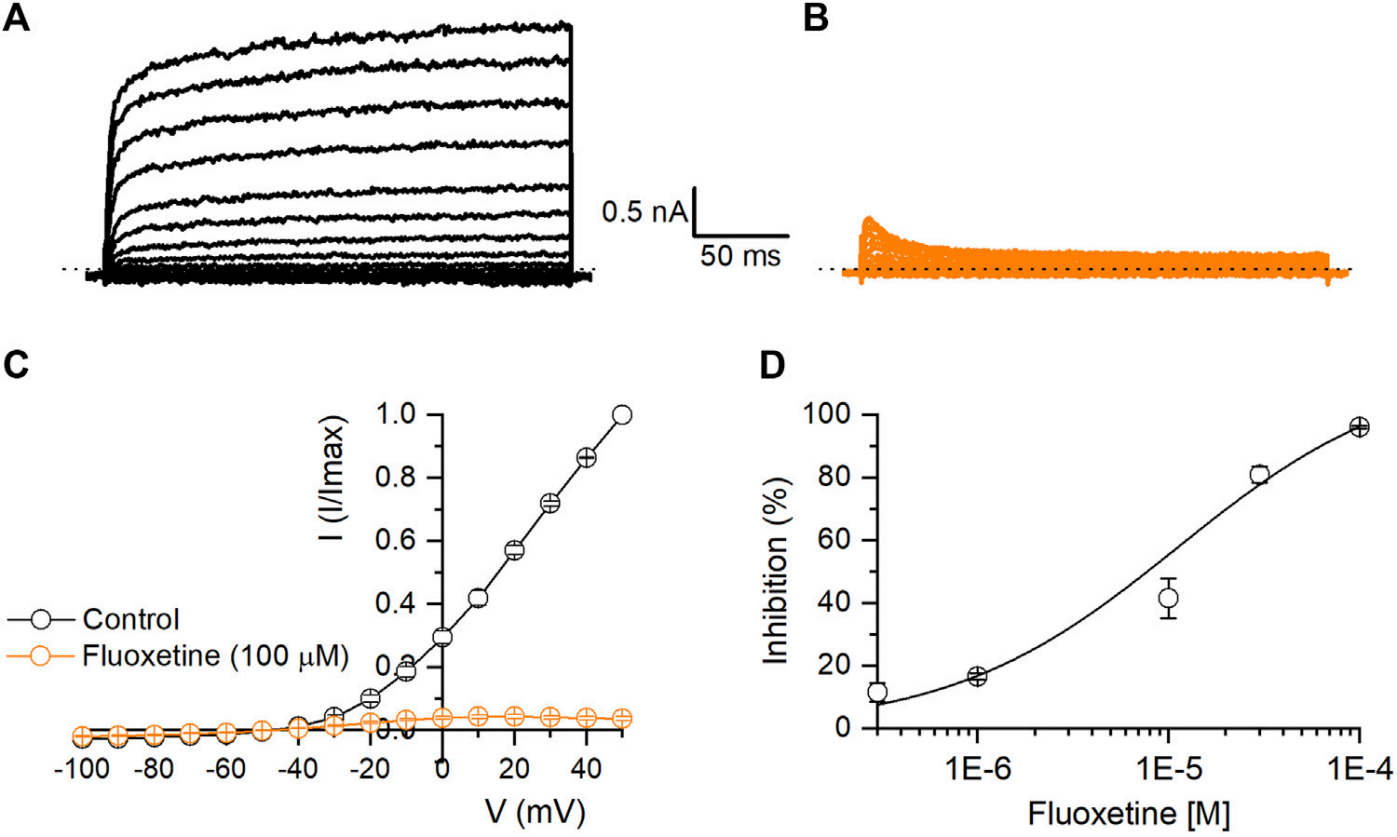
Inhibitory effect of fluoxetine on Kv10.1 channels. **(A,B)** Representative traces of a family of Kv10.1 currents in control (black) and in the presence of fluoxetine (100 μM; orange), respectively. **(C)** I-V relationship of Kv10.1 currents in control (black circles) and the presence of fluoxetine (100 μM; orange circles) (n = 5). **(D)** Dose-inhibition curves of fluoxetine on Kv10.1 currents (n ≥ 3 for each point). The solid line represents the fit to the Hill equation.

Fluoxetine is a selective serotonin reuptake inhibitor used as an antidepressant (Wille et al., 2008). Our results showed that fluoxetine induced a dose-dependent and reversible inhibition of Kv10.1 currents. At 100 μM, fluoxetine produced a 96.1% ± 2.9% inhibition of Kv10.1 currents (n = 5, *p* < 0.0001; paired *t*-test; see Figures 3A–C). Figure 3D shows the dose-concentration inhibition effect of Fluoxetine on Kv10.1 currents; these data were fitted with a Hill equation, which yielded an IC50 of 11.2 ± 6.2 µM and a Hill coefficient of 0.8 ± 0.1 (Figure 3D).

**FIGURE 4:**
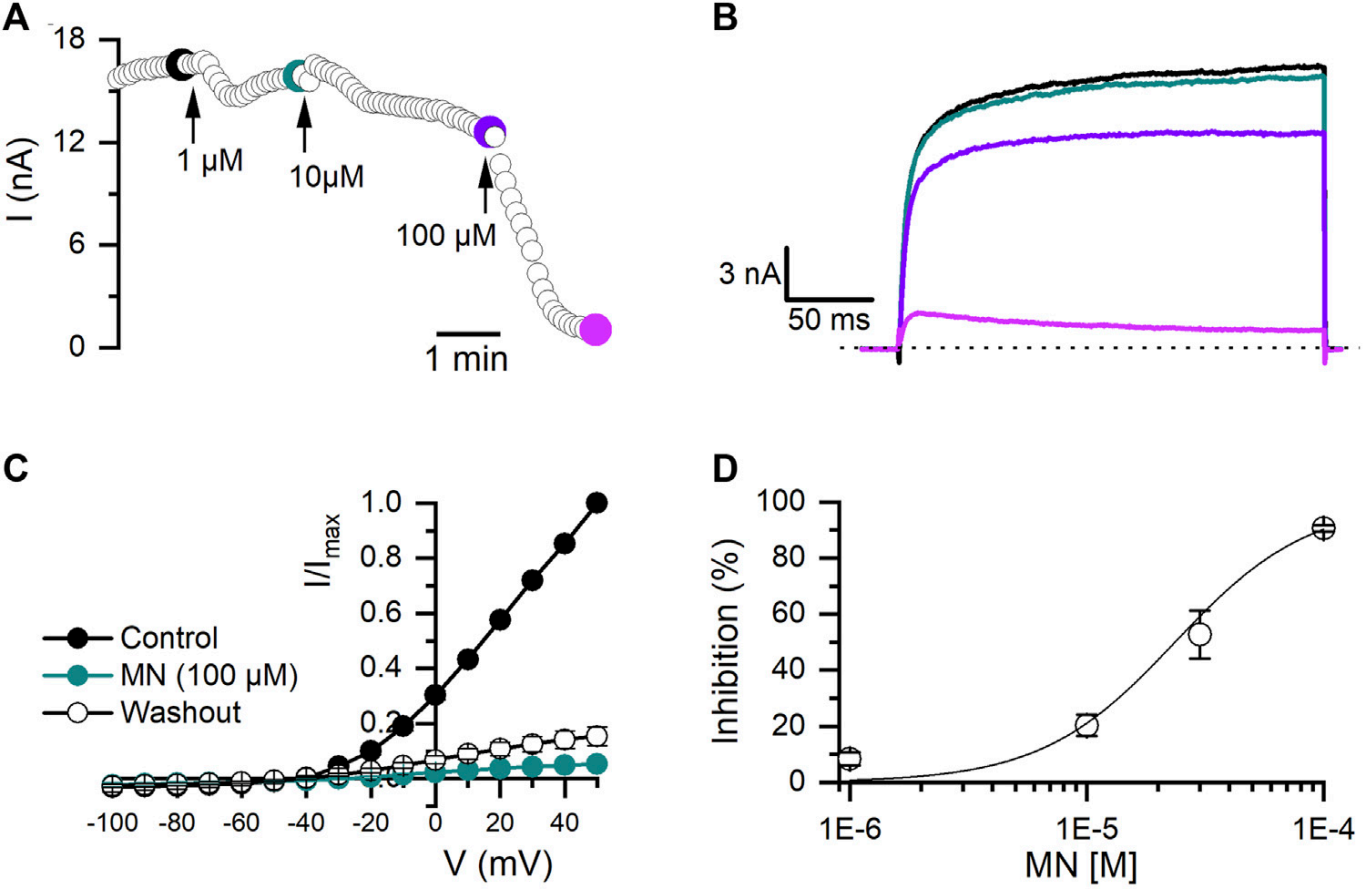
Effect of miconazole nitrate (MN) on Kv10.1 currents. **(A)** The temporal course of the inhibitory effect of MN (1, 10, and 100 µM) on Kv10.1 currents. The current traces for the time points marked with filled dots are presented in B. **(B)** Kv10.1 recordings in control (black) and in the presence of 1 (teal), 10 (purple), and 100 (pink) µM of MN **(C)** I-V relationship of Kv10.1 currents in control (black circles), the presence of MN (100 μM; teal circles), and during the washout (empty circles) (n = 5). **(D)** Dose-inhibition curves of MN on Kv10.1 currents (n ≥ 3 for each point). The solid line represents the fit to the Hill equation.

MN is an antifungal agent that has also been reported to have Kv blocker activity (Kikuchi et al., 2005). As shown in Figure 4A, MN produced a time and dose-dependent irreversible inhibition of Kv10.1 currents (Figure 4A). At 100 μM, MN inhibited Kv10.1 currents by 93% (n = 10, *p* < 0.0001; paired *t*-test) (Figure 4C). The inhibition percentage at each concentration tested were fitted with a Hill equation, which yielded an IC50 of 23.6 ± 5.0 µM and a Hill coefficient of 1.5 ± 0.3 (Figure 4D).

### 3.3 BL-1249 potentiates the activity of Kv10.1 channels

**FIGURE 5:**
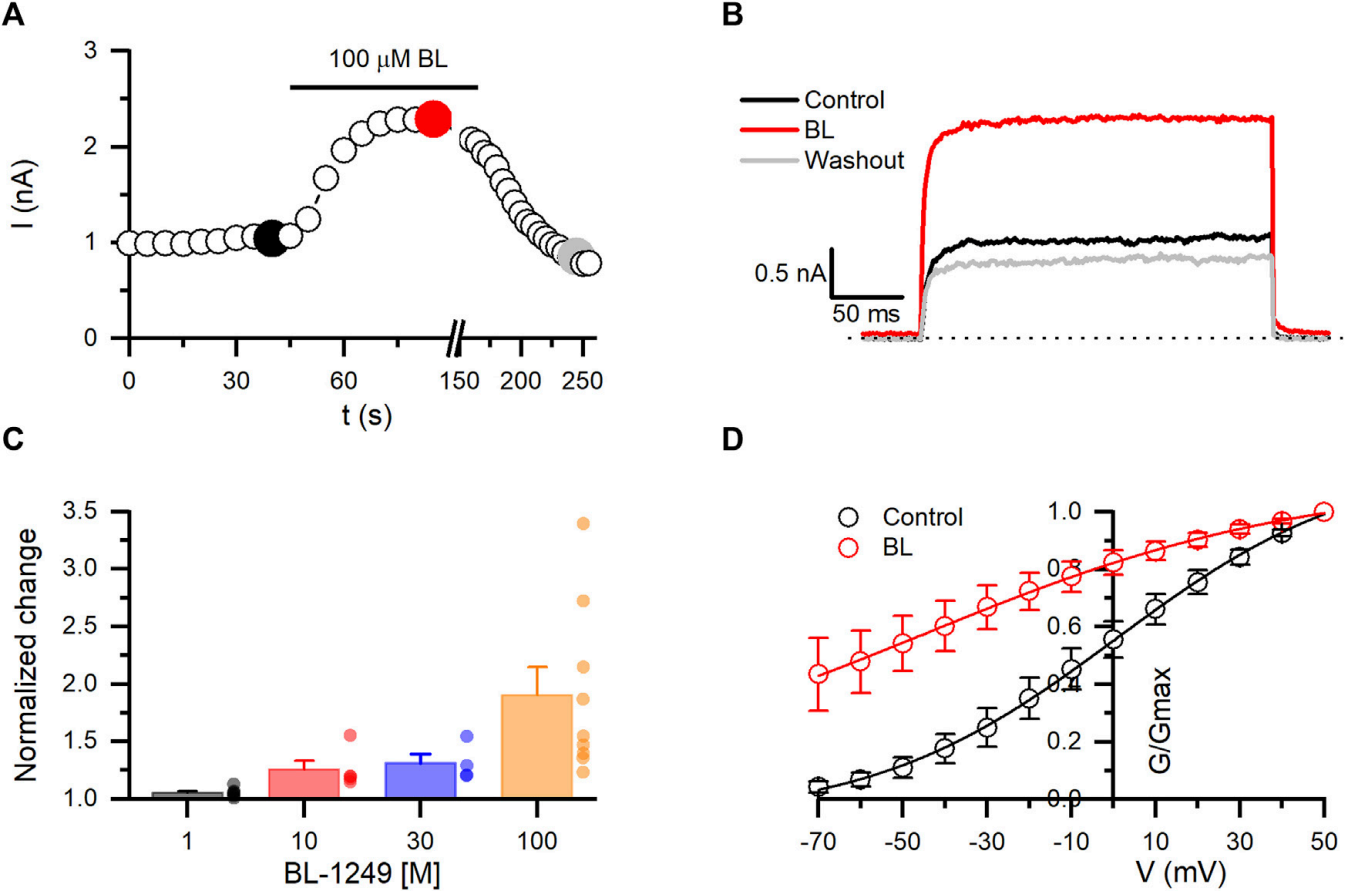
BL-1249 (BL) exhibits opener activity on Kv10.1 currents. **(A)** The temporal course of the opener effect of BL (100 µM) on Kv10.1 currents. The current traces for the time points marked with filled dots are presented in B. **(B)** Kv10.1 recordings in control (black), in the presence of BL (100 µM), and during the washout (gray). **(C)** Summary of the dose-dependent effect of BL on the Kv10.1 current. **(D)** Conductance-voltage relationship of the Kv10.1 channels in control and the presence of BL. The solid line represents the fit to the Boltzmann equation.

BL-1249 has been described as a negatively charged activator of different types of K^+^ channels (Schewe et al., 2019). Here, we observed that BL potentiates in a dose-dependent and reversible manner the Kv10.1 currents. Due to the solubility of BL, the maximum concentration tested was 100 µM. As shown in Figures 5A–C, rapidly increased by 90% the Kv10.1 currents measured at +30 mV (n = 9; *p* = 0.0001; paired *t*-test). Since we could not test higher concentrations of BL, the maximal potentiating concentration is unknown. However, we decided to perform a trend analysis to evaluate whether the dose-dependent effect followed a direct relationship (*p* = 0.0013; trendy test). Posterior, the dose-concentration fitting yielded an EC50 of 36.5 µM and a hill slope of 1.4. Analysis of Kv10.1 conductance showed that BL shifted their V_1/2_ activation by 44 mV to the left, from 0.6 ± 1.1 mV in control to −44.9 ± 6.2 mV in the presence of 100 µM BL (n = 6). These results suggest for the first time the potentiation effect of a molecule on Kv10.1 channels.

**FIGURE 6:**
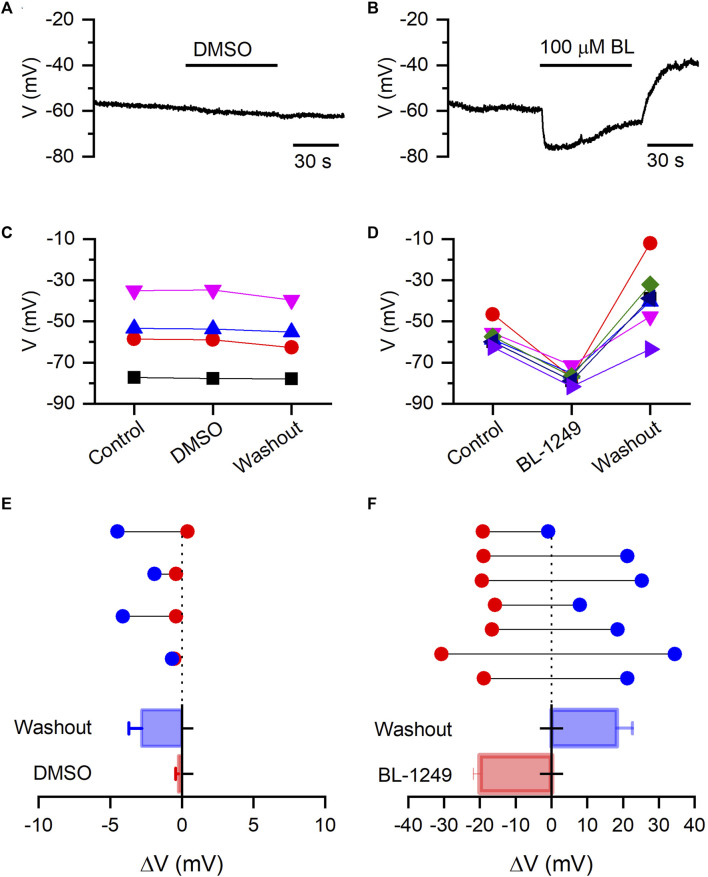
BL-1249 hyperpolarizes the resting membrane potential of HEK-Kv10.1 cells. **(A,B)** Current-clamp recording of RMP of HEK-Kv10.1 cells in the presence of DMSO and BL (100 µM), respectively. **(C,D)** Effects on the RMP of HEK-Kv10.1 cells recorded in the presence of DMSO (n = 4) and BL (n = 7), respectively. **(E,F)** Average effect of DMSO and BL on the RMP of HEK-Kv10.1 cells. Above, data represent the shift of RMP in each cell concerning the control condition (dotted line) obtained before applying every compound. Below is a summary histogram of the average change of the RMP.

Next, we are interested in evaluating whether BL could hyperpolarize the resting membrane potential (RMP) of HEK-Kv10.1 cells. We performed current-clamp recordings to measure the RMP without current injection. HEK-Kv10.1 cell had a RMP of −56.8 ± 3.1 mV (n = 11). In the presence of DMSO (applying for 1 min), the RMP did not show any statistically significant change, from −56.1 ± 8.7 mv in control to −56.3 ± 8.9 mV with DMSO (Figures 6A,C,E) (n = 4; *p* = 0.345; paired *t*-test). In contrast, BL (100 µM) hyperpolarized the RMP of HEK-Kv10.1 cells by 20 mV, from −57.3 ± 1.9 mV in control to −77.2 ± 1.2 mV in the presence of BL (Figures 6B,D,F) (n = 7; *p* < 0.0001; paired *t*-test). Interestingly, during the washout, the RMP suffered a transitory rebound to more depolarized values.

**FIGURE 7:**
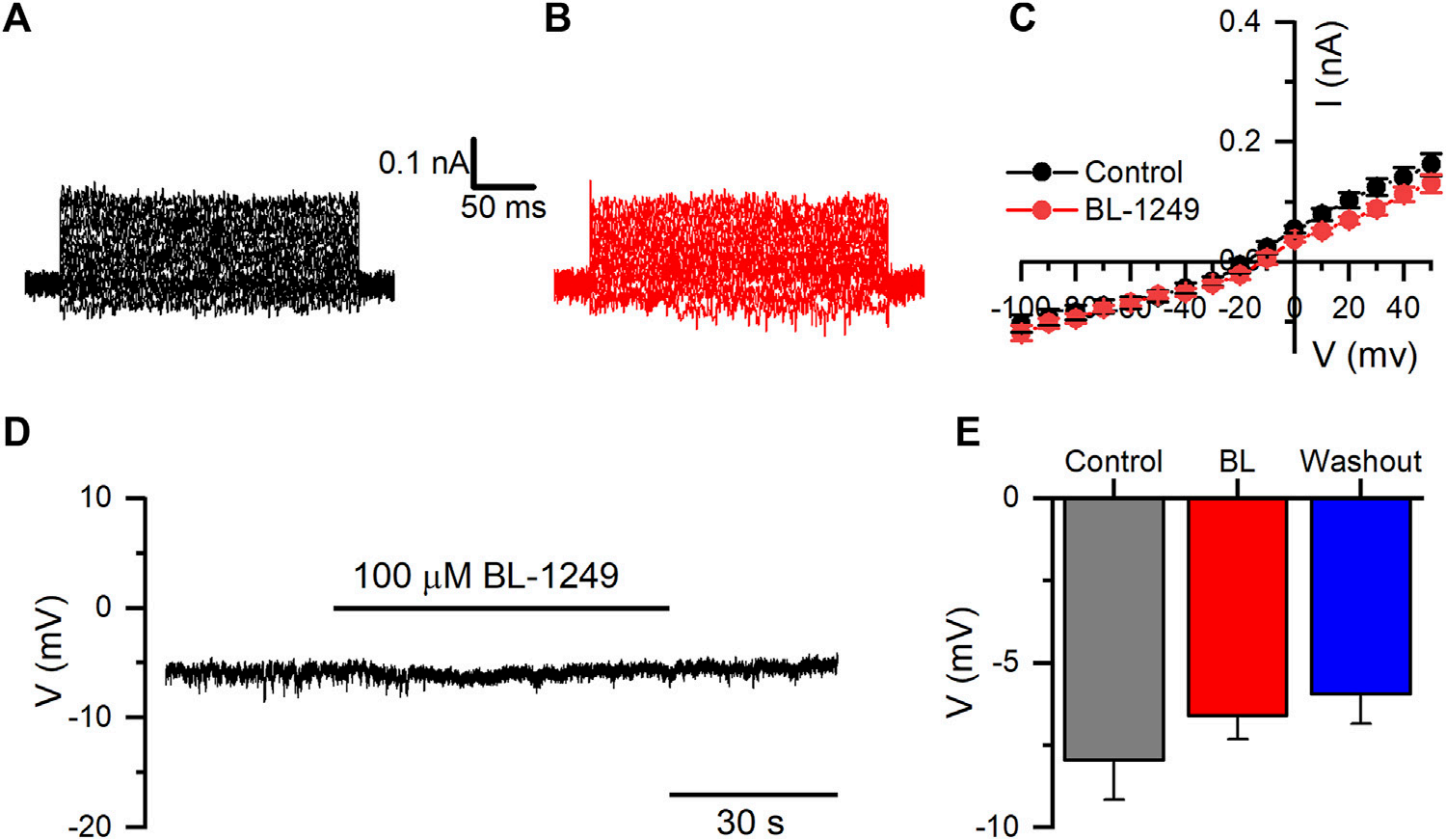
BL-1249 does not affect the endogenous potassium currents or RMP of HEK-WT cells. **(A, B)** representative family of endogenous K^+^ currents in HEK-WT cells in control and the presence of 100 μM BL-1249, respectively. **(C)** I-V relationship of HEK-WT currents in control (black circles) and the presence of BL-1249 (100 μM; red circles) (*n* = 10). **(D)** Representative current-clamp recording of RMP in HEK-WT cells in the presence of BL (100 μM). **(E)** The average effect of BL on the RMP of HEK-WT cells in control, in the presence of BL, and during the washout (*n* = 6).

We also evaluated whether BL could potentiate the activity of the endogenous currents of HEK-WT cells. BL did not show a significant effect on currents measured at +50 mV from 162.4 ± 18.4 pA in control to 131.2 ± 15.0 pA in the presence of 100 µM BL (n = 10; *p* = 114, paired *t*-test) (Figures 7A–C). Likewise, RMP in HEK-WT cells did not change when BL (100 µM) was added, yielding values from −7.9 ± 1.2 mV in control to −6.6 ± 0.7 mV with BL (n = 6; *p* = 1754, paired *t*-test) (Figures 7D, E).

**FIGURE 8:**
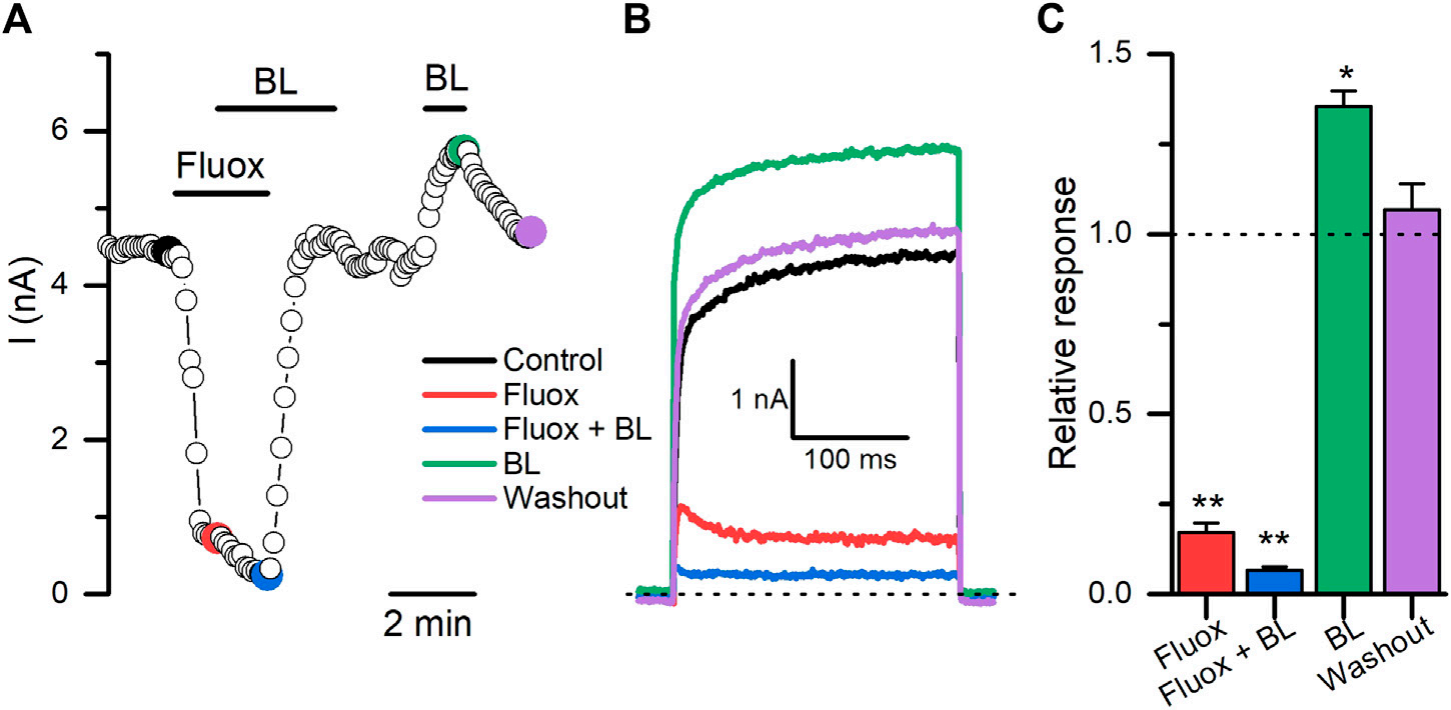
The opener activity of BL-1249 on Kv10.1 currents is obliterated in the presence of fluoxetine. **(A)** The time course of the activity of fluoxetine (30 μM; Fluox), fluoxetine plus BL-1249 (100 μM; BL), and BL on Kv10.1 currents. The current traces for the time points marked with filled dots are presented in B. **(B)** Kv10.1 recordings in control (black), in the presence of fluoxetine (red), fluoxetine plus BL (blue), BL (green), and during the washout (purple). **(C)** Summary of each treatment on Kv10.1 currents. Data are normalized to the control condition.

We tested next whether it was possible to counteract the inhibitory effect of some of the new antagonists described here with BL. As is shown in Figure 8, fluoxetine (30 µM) induced a rapid and statistically significant decrease (∼83%; n = 6; *p* = 0.0029, paired *t*-test) in the Kv10.1 currents measured at +30 mV. Surprisingly, the combined application of fluoxetine plus BL (100 µM) potentiated the inhibitory effect of fluoxetine to 93% (n = 6; *p* = 0.0034, paired *t*-test). The inhibitory effect of fluoxetine was reversible when the molecule was retired from the bath solution**.** Then, BL tested alone still could potentiate the Kv10.1 currents (*p* = 0.021, paired *t*-test). These results open new possibilities for pharmacological studies on the Kv10.1 channel.

## 4 Discussion

Ion channel pharmacology is essential to test and validate ion channel function in specific cells and tissues. In this way, ion channel research has been enriched with the increasing number of fluorescent dyes and the development of high-throughput platforms. Thus, cell-based fluorescence assays have become helpful for addressing ion channel research questions regarding the pharmacological characterization and discovery of known or unknown modulators, respectively (Vetter et al., 2020; Loza-Huerta et al., 2021).

Potassium channels coordinate multiple cellular phenomena and can be the origin of diverse pathologies (Prevarskaya et al., 2018; Taura et al., 2021). Consequently, potassium ion channels have emerged as targets with therapeutic opportunities (Bednenko et al., 2021; Mathie et al., 2021). Among them, the Kv10.1 channel has been widely studied, showing that their enriched expression in around 70% of cancer samples can confer malignant properties to cancer cells (Hemmerlein et al., 2006). Pharmacological evidence has validated Kv10.1’s role in different hallmarks of cancer, making them a promising target for drug discovery. Several Kv10.1-blockers have been described, but no one has shown sufficient selectivity by the channel (García-Quiroz and Camacho, 2011; Valdés-Abadía et al., 2019; Loza-Huerta et al., 2021; Toplak et al., 2022). This lack of selectivity is partly due to Kv10.1 showing high homology with Kv11.1 (HERG) potassium channels, so identifying a selective blocker has been challenging (Barros et al., 2020; Toplak et al., 2022).

Here, we studied whether it is possible to find new Kv10.1 modulators through a fluorescence assay associated with changes in membrane potential. The FMP reliability has been confirmed using small molecules previously described by our group as Kv10.1-inhibitors (Loza-Huerta et al., 2021). Here we observed that loperamide and amitriptyline decrease the fluorescence responses in HEK-Kv10.1 cells in the same proportion that astemizole, the classical Kv10.1-blocker. Experiments on HEK-WT cells showed that they also could produce changes in fluorescence in responses to 60 mM KCl; however, the amplitude of these responses was statistically smaller than HEK-Kv10.1 cells, indicating the contribution of Kv10.1. The more intense responses recorded in HEK-Kv10.1 cells can also be explained by their more hyperpolarized RMP than WT cells; thus, when cells are in the presence of 60 mM KCl, HEK-Kv10.1 suffer a higher depolarization, which could be directly proportional to fluorescence responses. Even if none of the molecules tested here are selective for Kv10.1 channels, the potent inhibitory effects of fluoxetine and MN constitute promising tools for further biophysical and molecular studies.

Fluoxetine, marketed as Prozac^®^, is used as an antidepressant by millions worldwide. This drug is a selective serotonin reuptake inhibitor (Wille et al., 2008). Fluoxetine also exerts other effects, including inhibiting diverse ion channels (Thomas et al., 2002; Min et al., 2008; Jeong et al., 2013). The present work reveals that fluoxetine is a potent inhibitor of the Kv10.1 channels, with an IC50 value of 11 μM, an IC50 close to that of other Kv10.1-blocker described previously. Although Kv10.1-pharmacology has been focused on cancer biology, the results suggest that fluoxetine perhaps also affects Kv10.1 channels expressed in the brain. Pharmacodynamic studies in patients have estimated that fluoxetine can reach steady-state serum concentrations ranging from 0.3 to 2.6 µM and brain tissue concentrations ranging from 5 to 17 µM (Bolo et al., 2000), the last concentrations in a range close to the IC50 described here for Kv10.1. It is uncertain whether some side effects of chronic use of fluoxetine can be related to Kv10.1 inhibition. Fluoxetine is also described as a Kv11.1-blocker with an IC50 of 3.1 µM, providing the molecular mechanism through which fluoxetine prolongs the electrocardiographic QT interval in patients who use this drug (Thomas et al., 2002). Given the high homology among Kv10.1 and Kv11.1 channels, it is not surprising that fluoxetine exerts inhibitory effects on both ion channels.

**FIGURE 9:**
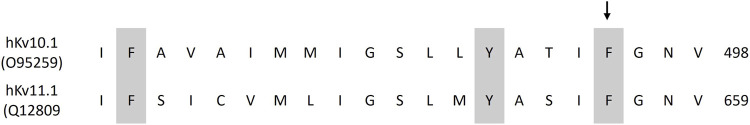
Alignment of the S6 segment of the human Kv10.1 and Kv11.1 channels. Conserved aromatic amino acids are shaded in gray. Sequence numbering is shown on the right. Arrow indicates the position of residue F656 in Kv11.1.

Also, miconazole nitrate exerted a dose-dependent inhibition on Kv10.1 activity. MN is a drug used to treat fungal infections and can be administered vaginally, orally, or parenterally (Al-Badr, 2005). As well as fluoxetine, MN has been described previously as a HERG-blocker with an IC50 of 2.1 µM (Kikuchi et al., 2005), 11 times more potent than the IC50 described here for Kv10.1. Moreover, it has been reported that MN binds to the aromatic residue F656 in the S6 of the HERG channel (Kikuchi et al., 2005). Alignment of the S6 sequence of Kv11.1 and Kv10.1 channels demonstrated that the F656 residue is conserved in the position F495 of Kv10.1 channels (Figure 9), suggesting that MN can inhibit Kv10.1 through a similar mechanism than in Kv11.1 channels. Nevertheless, mutagenesis experiments of Kv10.1 are needed to test this hypothesis.

Most of the pharmacological research on Kv10.1 has focused on looking for inhibitors. Common sense said that if Kv10.1 overexpression offers advantages to cancer cells, the inhibition of the channel would have positive results reducing several hallmarks of cancer cells, and currently, this has happened. However, a new direction in the pharmacology of the K^+^ channel is the use of openers that control cellular excitability in neuronal pathologies (Lawson, 2000; Judge et al., 2007) or pathologies associated with changes in the resting membrane potential, including cancer (Fukushiro-Lopes et al., 2018).

Our study shows, for the first time, that BL-1249 enhances the Kv10.1 activity and that it can hyperpolarize the resting membrane potential of cells expressing this channel; these effects were absent when BL-1249 (at the highest concentration tested) was applied to wild-type HEK293 cells, indicating a direct consequence of BL-1249 on Kv10.1 channels. BL-1249 is a non-steroidal anti-inflammatory, which has been described as an activator of K^+^ channels of the K2P, TREK-1, and TREK-2 type, showed an EC50 of ∼5 µM and ∼8 μM, respectively (Iwaki et al., 2019). Here, the EC50 for BL on the Kv10.1 channel was five times higher. K^+^ channels have evolved to respond to multiple stimuli (voltage, temperature, second messengers, *etc.*), so it is thought that their pharmacological activation may follow common mechanisms. In the case of negatively charged activators, such as BL-1249, it has been reported that these molecules bind below the selectivity filter, where the charge of the molecules promotes K^+^ binding to the pore cavity, which causes an increase in K^+^ occupying the selectivity filter, which finally increases ion permeation and channel-open probability (Schewe et al., 2019). This phenomenon was observed in the Kv11.1 channel (Schewe et al., 2019) and because Kv10.1 presents structural similarities with Kv11.1 (Whicher and MacKinnon, 2016; Wang and MacKinnon, 2017; Barros et al., 2020), we can hypothesize that the opener effect of BL-1249 is through the same molecular mechanism; however, more experiments are needed to answer this question. Remarkably, the opener activity of BL on Kv10.1 was counteracted when BL was added in the presence of fluoxetine; instead, the inhibitory effect of fluoxetine was potentiated by BL application. More detailed experiments are needed to determine how both molecules interact on the Kv10.1 channel to produce this effect and whether this also could be observed with other molecules acting as gating modifiers or pore blockers of Kv10.1.

The potassium channels’ activity is associated with excitability processes in neurons and myocytes (Taura et al., 2021), while in non-excitable cells, they seem to participate in the cell cycle and proliferation (Abdul Kadir et al., 2018). In this sense, cancer cells have more depolarized RMP than their parental normal and quiescent cells, indicating that transmembrane voltage could be associated with pro-proliferation signals (Zhou et al., 2015; Urrego et al., 2017; Abdul Kadir et al., 2018). However, molecular mechanisms are poorly understood and raise the question about the role of potassium channels in cancer proliferation. Recently, it has been reported that membrane potential depolarization induces changes in the organization of phospholipids in the inner left of the membrane, increasing K-Ras nanoclusters and enhancing the K-Ras-dependent MAPK signaling (Zhou et al., 2015). In contrast, the membrane potential hyperpolarization disrupts this pathway.

In this sense, the Kv10.1 channel shows an increased expression in various types of tumors (Hemmerlein et al., 2006), and this elevated expression has been associated with different stages of the cell cycle (Urrego et al., 2014; Sánchez et al., 2016), so that the pharmacological manipulation of this channel, using openers would favor the hyperpolarization of the membrane potential of tumor cells, an effect observed during our experiments, and which can slow down cell proliferation of tumor cells expressing the Kv10.1 channel. Additionally, it can be hypothesized that the hyperpolarization due to the Kv10.1 activation would decrease the activity of voltage-gated calcium channels (also involved in tumorigenesis) (Prevarskaya et al., 2018), decreasing intracellular calcium concentration, which plays a crucial role in carcinogenesis. Promising results using the molecule NS-1643, also an opener of Kv11.1 channels, have shown positive results in breast cancer (Lansu and Gentile, 2013; Fukushiro-Lopes et al., 2018). In summary, FMP assays are an effective and reliable method for the primary screening of different molecules with potential activity on Kv10.1 channels.

## Data Availability

The raw data supporting the conclusions of this article will be made available by the authors, without undue reservation.

## References

[B1] Abdul Kadir L. Stacey M. Barrett-Jolley R. (2018). Emerging roles of the membrane potential: Action beyond the action potential. Front. Physiol. 9, 1661. 10.3389/fphys.2018.01661 30519193 PMC6258788

[B2] Al-Badr A. A. (2005). Miconazole nitrate: Comprehensive profile. Profiles Drug Subst. Excipients Relat. Methodol. 32, 3–65. 10.1016/S0099-5428(05)32001-6 22469081

[B3] Bai Y. Liao H. Liu T. Zeng X. Xiao F. Luo L. (2013). MiR-296-3p regulates cell growth and multi-drug resistance of human glioblastoma by targeting ether-à-go-go (EAG1). Eur. J. Cancer 49, 710–724. 10.1016/j.ejca.2012.08.020 22999387

[B4] Barros F. de la Peña P. Domínguez P. Sierra L. M. Pardo L. A. (2020). The EAG voltage-dependent K+ channel subfamily: Similarities and differences in structural organization and gating. Front. Pharmacol. 11, 411–419. 10.3389/fphar.2020.00411 32351384 PMC7174612

[B5] Bednenko J. Colussi P. Hussain S. Zhang Y. Clark T. (2021). “Therapeutic antibodies targeting potassium ion channels,” in Handbook of experimental pharmacology (Berlin: Springer). 10.1007/164_2021_464 33963460

[B6] Bolo N. R. Hodé Y. Nédélec J. F. Lainé E. Wagner G. MacHer J. P. (2000). Brain pharmacokinetics and tissue distribution *in vivo* of fluvoxamine and fluoxetine by fluorine magnetic resonance spectroscopy. Neuropsychopharmacology 23, 428–438. 10.1016/S0893-133X(00)00116-0 10989270

[B7] Curtis H. J. Cole K. S. (1942). Membrane resting and action potentials from the squid giant axon. J. Cell. Comp. Physiol. 19, 135–144. 10.1002/jcp.1030190202

[B8] Downie B. R. Sánchez A. Knötgen H. Contreras-Jurado C. Gymnopoulos M. Weber C. (2008). Eag1 expression interferes with hypoxia homeostasis and induces angiogenesis in tumors. J. Biol. Chem. 283, 36234–36240. 10.1074/jbc.M801830200 18927085 PMC2606018

[B9] Farias L. M. B. Ocaña D. B. Díaz L. Larrea F. Avila-Chávez E. Cadena A. (2004). Ether à go-go potassium channels as human cervical cancer markers. Cancer Res. 64, 6996–7001. 10.1158/0008-5472.CAN-04-1204 15466192

[B10] Fukushiro-Lopes D. F. Hegel A. D. Rao V. Wyatt D. Baker A. Breuer E. K. (2018). Preclinical study of a Kv11.1 potassium channel activator as antineoplastic approach for breast cancer. Oncotarget 9, 3321–3337. 10.18632/oncotarget.22925 29423049 PMC5790466

[B11] García-Quiroz J. Camacho J. (2011). Astemizole: An old anti-histamine as a new promising anticancer drug. Anticancer. Agents Med. Chem. 11, 307–314. 10.2174/187152011795347513 21443504

[B12] Hemmerlein B. Weseloh R. M. de Queiroz F. M. Knötgen H. Sánchez A. Rubio M. E. (2006). Overexpression of Eag1 potassium channels in clinical tumours. Mol. Cancer 5, 41. 10.1186/1476-4598-5-41 17022810 PMC1621079

[B13] Iwaki Y. Yashiro K. Kokubo M. Mori T. Wieting J. M. McGowan K. M. (2019). Towards a TREK-1/2 (TWIK-Related K+ Channel 1 and 2) dual activator tool compound: Multi-dimensional optimization of BL-1249. Bioorg. Med. Chem. Lett. 29, 1601–1604. 10.1016/j.bmcl.2019.04.048 31072652

[B14] Jeong I. Choi J. S. Hahn S. J. (2013). Effects of fluoxetine on cloned Kv4.3 potassium channels. Brain Res. 1500, 10–18. 10.1016/j.brainres.2013.01.028 23348380

[B15] Judge S. I. V. Smith P. J. Stewart P. E. Bever C. T. (2007). Potassium channel blockers and openers as CNS neurologic therapeutic agents. Recent Pat. CNS Drug Discov. 2, 200–228. 10.2174/157488907782411765 18221232

[B16] Kikuchi K. Nagatomo T. Abe H. Kawakami K. Duff H. J. Makielski J. C. (2005). Blockade of HERG cardiac K + current by antifungal drug miconazole. Br. J. Pharmacol. 144, 840–848. 10.1038/sj.bjp.0706095 15778703 PMC1576066

[B17] Lawson K. (2000). Potassium channel openers as potential therapeutic weapons in ion channel disease. Kidney Int. 57, 838–845. 10.1046/j.1523-1755.2000.00923.x 10720937

[B18] Lansu K. Gentile S. (2013). Potassium channel activation inhibits proliferation of breast cancer cells by activating a senescence program. Cell Death Dis. 4, e652–e659. 10.1038/cddis.2013.174 23744352 PMC3698542

[B19] Loza-Huerta A. Milo E. Picones A. Hernández-Cruz A. Luis E. (2021). Thallium-sensitive fluorescent assay reveals loperamide as a new inhibitor of the potassium channel Kv10.1. Pharmacol. Rep. 73, 1744–1753. 10.1007/s43440-021-00304-5 34213738

[B20] Luis E. Anaya-Hernández A. León-Sánchez P. Durán-Pastén M. L. (2022a). The Kv10.1 channel: A promising target in cancer. Int. J. Mol. Sci. 23, 8458. 10.3390/ijms23158458 35955591 PMC9369319

[B21] Luis E. Lara Figueroa C. O. Durán Pastén M. L. Azorín Vega E. P. (2022b). Role of gamma radiation on functional expression of the voltage-gated potassium channel Kv10.1 and its importance in the radiobiological response. Appl. Radiat. Isot. 187, 110331. 10.1016/j.apradiso.2022.110331 35764005

[B22] Martínez R. Stühmer W. Martin S. Schell J. Reichmann A. Rohde V. (2015). Analysis of the expression of Kv10.1 potassium channel in patients with brain metastases and glioblastoma multiforme: Impact on survival. BMC Cancer 15, 839. 10.1186/s12885-015-1848-y 26530050 PMC4632660

[B23] Mathie A. Veale E. L. Golluscio A. Holden R. G. Walsh Y. (2021). “Pharmacological approaches to studying potassium channels,” in Handbook of experimental pharmacology (Berlin: Springer). 10.1007/164_2021_502 34195873

[B24] Min J. S. Hye S. A. Sang J. H. Bok H. C. (2008). Open channel block of Kv3.1 currents by fluoxetine. J. Pharmacol. Sci. 106, 38–45. 10.1254/jphs.FP0070759 18187934

[B25] Neher E. Sakmann B. (1976). Single-channel currents recorded from membrane of denervated frog muscle fibres. Nature 260, 799–802. 10.1038/260799a0 1083489

[B26] Ortiz C. S. Montante-Montes D. Saqui-Salces M. Hinojosa L. M. Gamboa-Dominguez A. Hernández-Gallegos E. (2011). Eag1 potassium channels as markers of cervical dysplasia. Oncol. Rep. 26, 1377–1383. 10.3892/or.2011.1441 21887469

[B27] Pardo L. A. Del Camino D. Sánchez A. Alves F. Brüggemann A. Beckh S. (1999). Oncogenic potential of EAG K+ channels. EMBO J. 18, 5540–5547. 10.1093/emboj/18.20.5540 10523298 PMC1171622

[B28] Prevarskaya N. Skryma R. Shuba Y. (2018). Ion channels in cancer: Are cancer hallmarks oncochannelopathies? Physiol. Rev. 98, 559–621. 10.1152/physrev.00044.2016 29412049

[B29] Sánchez A. Urrego D. Pardo L. A. (2016). Cyclic expression of the voltage‐gated potassium channel K V 10.1 promotes disassembly of the primary cilium. EMBO Rep. 17, 708–723. 10.15252/embr.201541082 27113750 PMC5341513

[B30] Schewe M. Sun H. Mert Ü. Mackenzie A. Pike A. C. W. Schulz F. (2019). A pharmacological master key mechanism that unlocks the selectivity filter gate in K + channels. Sci. (80) 363, 875–880. 10.1126/science.aav0569 PMC6982535 30792303

[B31] Taura J. Kircher D. M. Gameiro-Ros I. Slesinger P. A. (2021). “Comparison of K+ channel families,” in Handbook of experimental pharmacology (Berlin: Springer), 1–49. 10.1007/164_2021_460 34247281

[B32] Thomas D. Gut B. Wendt-Nordahl G. Kiehn J. (2002). The antidepressant drug fluoxetine is an inhibitor of human ether-a-go-go-related gene (HERG) potassium channels. J. Pharmacol. Exp. Ther. 300, 543–548. 10.1124/jpet.300.2.543 11805215

[B33] Toplak Ž. Hendrickx L. A. Abdelaziz R. Shi X. Peigneur S. Tomašič T. (2022). Overcoming challenges of HERG potassium channel liability through rational design: Eag1 inhibitors for cancer treatment. Med. Res. Rev. 42, 183–226. 10.1002/med.21808 33945158

[B34] Urrego D. Sánchez A. Tomczak A. P. Pardo L. A. (2017). The electric fence to cell-cycle progression: Do local changes in membrane potential facilitate disassembly of the primary cilium? BioEssays 39, 1600190. 10.1002/bies.201600190 28370099

[B35] Urrego D. Tomczak A. P. Zahed F. Stühmer W. Pardo L. A. (2014). Potassium channels in cell cycle and cell proliferation. Philos. Trans. R. Soc. B Biol. Sci. 369, 20130094–20130099. 10.1098/rstb.2013.0094 PMC3917348 24493742

[B36] Valdés-Abadía B. Morán-Zendejas R. Rangel-Flores J. M. Rodríguez-Menchaca A. A. (2019). Chloroquine inhibits tumor-related Kv10.1 channel and decreases migration of MDA-MB-231 breast cancer cells *in vitro* . Eur. J. Pharmacol. 855, 262–266. 10.1016/j.ejphar.2019.05.017 31082369

[B37] Verkhratsky A. Parpura V. (2014). History of electrophysiology and the patch clamp. Methods Mol. Biol. 1183, 1–19. 10.1007/978-1-4939-1096-0_1 25023299

[B38] Vetter I. Carter D. Bassett J. Deuis J. R. Tay B. Jami S. (2020). “High-throughput fluorescence assays for ion channels and GPCRs,” in Advances in experimental medicine and biology (Berlin: Springer), 27–72. 10.1007/978-3-030-12457-1_3 31646506

[B39] Wang W. MacKinnon R. (2017). Cryo-EM structure of the open human ether-à-go-go-related K+ channel hERG. Cell 169, 422–430.e10. 10.1016/j.cell.2017.03.048 28431243 PMC5484391

[B40] Whicher J. R. MacKinnon R. (2016). Structure of the voltage-gated K⁺ channel Eag1 reveals an alternative voltage sensing mechanism. Sci. (80) 353, 664–669. 10.1126/science.aaf8070 PMC5477842 27516594

[B41] Wille S. M. R. Cooreman S. G. Neels H. M. Lambert W. E. E. (2008). Relevant issues in the monitoring and the toxicology of antidepressants. Crit. Rev. Clin. Lab. Sci. 45, 25–89. 10.1080/10408360701713112 18293180

[B42] Zhou Y. Wong C. O. Cho K. J. Van Der Hoeven D. Liang H. Thakur D. P. (2015). SIGNAL TRANSDUCTION. Membrane potential modulates plasma membrane phospholipid dynamics and K-Ras signaling. Sci. (80-. ) 349, 873–876. 10.1126/science.aaa5619 PMC4687752 26293964

